# ﻿Discovery of the genus *Platycotylus* Olliff, 1883 (Coleoptera, Tenebrionidae) in Japan: Description of a new and remarkable species

**DOI:** 10.3897/zookeys.1076.75846

**Published:** 2021-12-09

**Authors:** Takahiro Yoshida1, Kiyoshi Ando2

**Affiliations:** 1 Systematic Zoology Laboratory, Department of Biological Sciences, Graduate School of Science, Tokyo Metropolitan University, 1–1 Minami-osawa, Hachioji, Tokyo, 192–0397, Japan Tokyo Metropolitan University Hachioji Japan; 2 Entomological Laboratory, Faculty of Agriculture, Ehime University, Tarumi 3–5–7, Matsuyama, 790–8566, Japan Ehime University Matsuyama Japan

**Keywords:** Epistomal horn, Nakanoshima Island, Palorini, taxonomy, Tokara Islands, twisted aedeagus

## Abstract

The genus *Platycotylus* Olliff, 1883 (Coleoptera: Tenebrionidae) is recorded from Japan (Nakanoshima Island, Tokara Islands) for the first time, through the discovery of a new and remarkable species, *Platycotylusmerkli***sp. nov.**, which is described herein. The male of this new species can be distinguished from all known males of other congeneric species by its long and asymmetrical epistomal horn. Although this new species is most similar to *Platycotylusparvicollis* (Pic, 1923), for which a male has not been examined, it can be distinguished from this species by its simple sparse pronotal punctation, smaller eyes, and acutely produced temples.

## ﻿Introduction

The tribe Palorini Matthews, 2003 was initially proposed by Matthews (2003a) as a new subfamily including 10 genera: *Platycotylus* Olliff, 1883, *Eutermicola* Lea, 1916, *Ulomotypus* Broun, 1886, and the seven genera of “the *Palorus* genus group” of Halstead (1967), namely *Astalbus* Fairmaire, 1900, *Austropalorus* Halstead, 1967, *Palorinus* Blair, 1930, *Palorus* Mulsant, 1854, *Prolabrus* Fairmaire, 1897, *Pseudeba* Blackburn, 1903, and *Ulomina* Baudi di Selve, 1876. Later, it was downgraded to the rank of tribe by Bouchard et al. (2005), which was confirmed by subsequent authors (e.g., Matthews and Bouchard 2008; Matthews et al. 2010). Two additional palorine genera were later described: the extant genus *Paloropsis* Masumoto & Grimm, 2004 from Japan and the extinct genus *Vabole* Alekseev & Nabozhenko, 2015 from Eocene Baltic amber. Except for several cosmopolitan pest species of stored products, most species of Palorini are absent from the New World (Matthews and Bouchard 2008). Some genera are endemic to principal areas, i.e., two, three, and one genera are endemic to Madagascar, northern Australia, and New Zealand, respectively, suggesting that the ancestor of this tribe occurred in Gondwana before it broke up (Matthews and Bouchard 2008; Alekseev and Nabozhenko 2017).

The genus *Platycotylus* was established within the family Cucujidae by Olliff (1883) based on a single species, *Platycotylusinusiatus* Olliff, 1883. Doyen (1984) added *Doliemanitidula* Macleay, 1872 (= *Platycotylusnitidulus*) to this genus and implied that this genus was probably closely related to the genus *Lorelus* Sharp, 1876 (Tenebrionidae: Lagriinae). Subsequently, *Platycotylus* was finally assigned to the family Tenebrionidae and placed within the tribe Triboliini Gistel, 1848 (Tenebrioninae) by Doyen et al. (1990). Matthews (2003a, 2003b) included *Platycotylus* in the taxon currently regarded as Palorini (as presented above).

*Platycotylus* is composed of five species that are widespread in Africa, Southeast Asia, and Australia (Merkl 1992; Schawaller 2014). Two species, *Platycotylusferrugineus* Kaszab, 1939 and *P.nitidulus*, have been recorded in Taiwan (Ando et al. 2016). All species have flattened bodies and are believed to live under hardly loosened bark of dead trees like laemophloeids, silvanids, and salpingids (Schawaller 2014; Alekseev and Nabozhenko 2017). In this study, we describe a new and morphologically remarkable species, *Platycotylusmerkli* sp. nov., from Nakanoshima Island, Tokara Islands, Japan, which represents the first record of this genus in Japan.

## ﻿Materials and methods

### ﻿Morphology, dissection and photographic techniques

**Figure 1.**
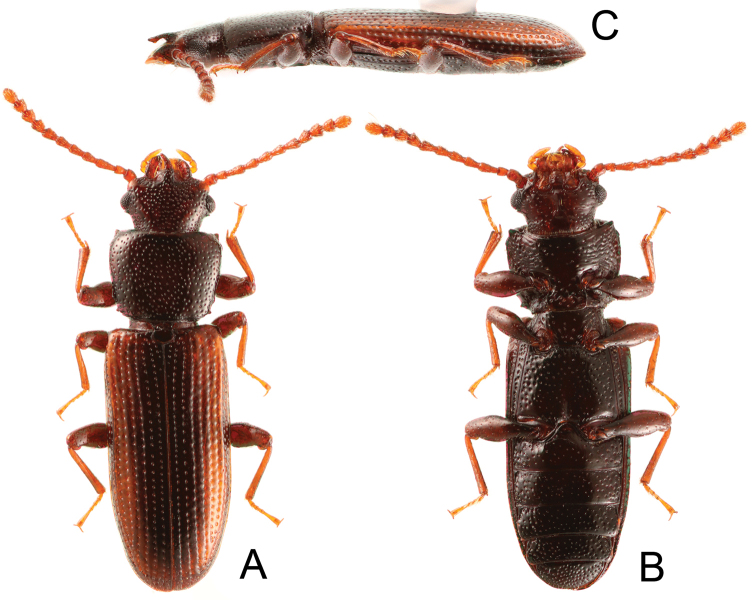
Habitus of *Platycotylusmerkli* sp. nov., holotype, male **A** Dorsal **B** ventral **C** lateral.

Observations of external characteristics and dissections were conducted using stereoscopic microscopes (Nikon SMZ1500 or Leica MZ16). The habitus images of the holotype (Fig. 1) were taken with a digital camera (Canon EOS 7D) fitted with a macro-objective (MP-E 65 mm) before dissection, and composite images of adults were produced using the automontage software Combine ZM and completed in Photoshop 6.0 (Adobe Systems Inc.). The abdomen of the holotype was removed and soaked in a 10% potassium hydroxide solution at room temperature overnight. After it was rinsed in water, the soaked abdomen was dissected under a stereoscopic microscope (Nikon SMZ1500) using fine insect pins; specifically, the male genital organs were detached for observation. The dissected parts were mounted in Euparal on a slide and observed under an optical microscope (Nikon Eclipse E400). After observations were completed, the dissected genitalia and other abdominal segments were mounted in Euparal on a glass coverslip glued to a piece of cardboard and pinned with the relevant specimen.

### ﻿Terminology, abbreviations and specimen deposition

Morphological terminology follows Matthews and Bouchard (2008). Examined specimens were deposited in the Ehime University Museum, Matsuyama, Japan (**EUMJ**).

## ﻿Taxonomy

### Order Coleoptera

**Family Tenebrionidae**

**Tribe Palorini**

#### ﻿ Platycotylus merkli sp. nov.

Taxon classificationAnimaliaColeopteraTenebrionidae

2FA1285C-7F2D-52E6-8EE7-1883AA3570E8

http://zoobank.org/324CF110-BE9C-400A-B497-AAA134298431

**Figure 2.**
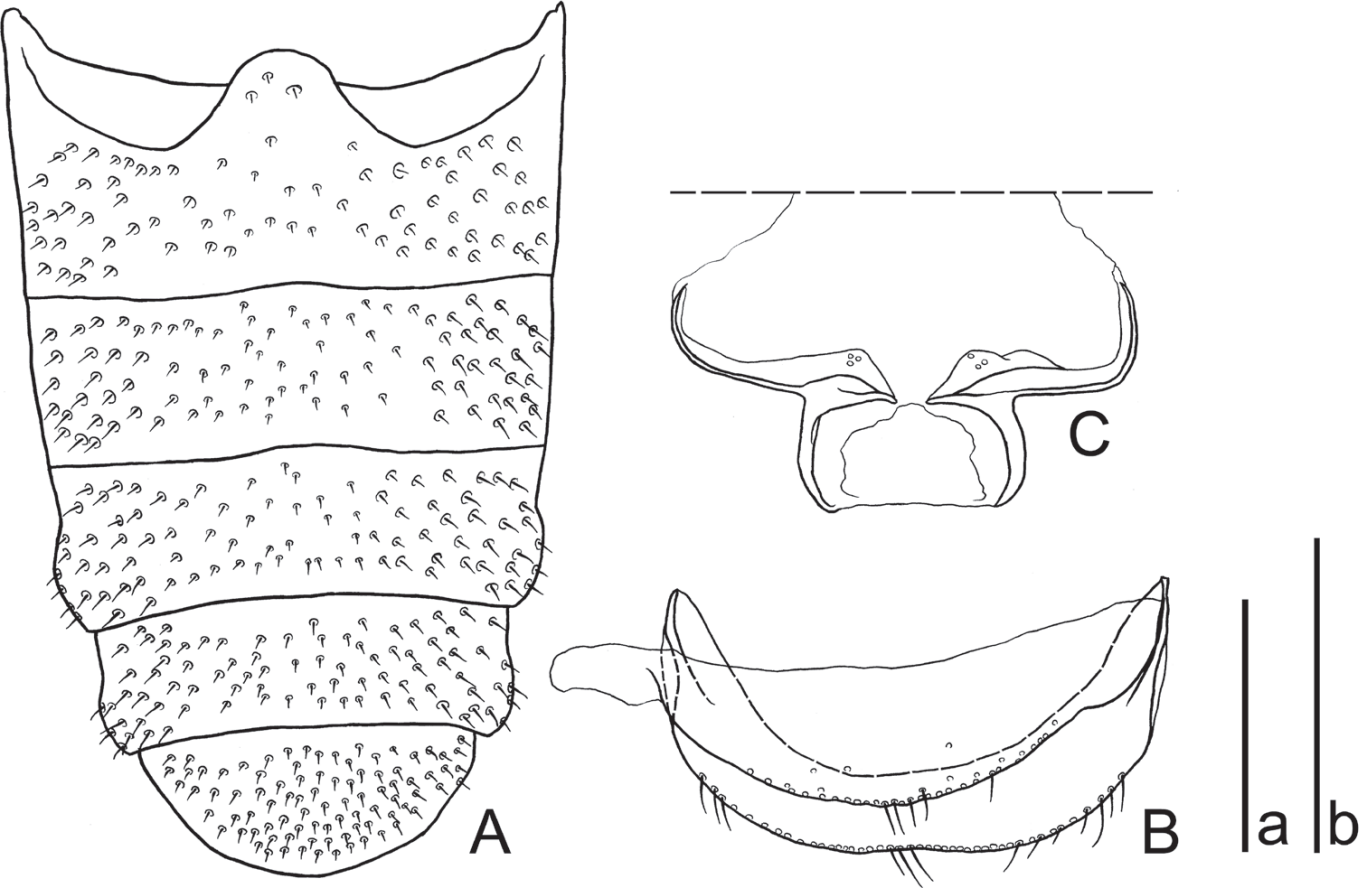
Abdominal segments of *Platycotylusmerkli* sp. nov., holotype, male **A** Ventrites **B** 8^th^ segment, ventral view **C** 9^th^ sternite, ventral view. Scale bars: 1.0 mm **a** (**A**); 0.5 mm (**B, C**).

**Figure 3.**
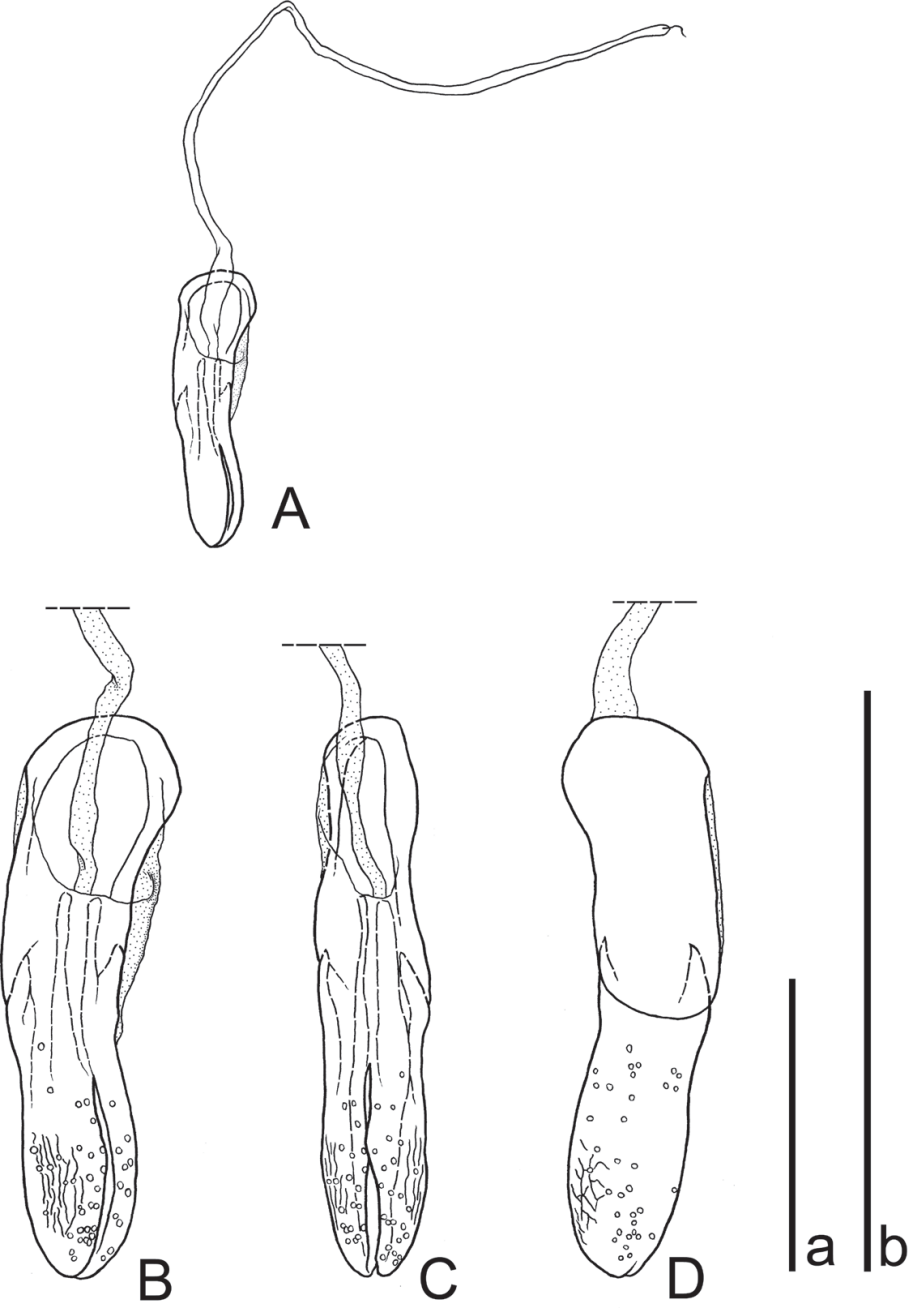
Aedeagus of *Platycotylusmerkli* sp. nov., holotype **A, B** Dorsal **C** dorsolateral **D** ventral. Scale bars: 0.5 mm **a** for **A**; **b** for **B–D**.

Figures 1 , 2 , 3 Japanese name: Tsuji-hirata-hime-kokunusutomodoki

##### Type specimen.

***Holotype***: ♂, Japan, Kagoshima Prefecture, Toshima Village, Tokara Islands, Tokara-Nakanoshima Island, Nanatsuyama, 7.VII.2019, leg. Naomichi Tsuji, “under permission” (EUMJ).

##### Differential diagnosis.

According to Merkl (1992), the epistome structures of males are important diagnostic characteristics for *Platycotylus* species. All known *Platycotylus* males present a pair of short tubercles at the middle of the epistome, except for *Platycotyluspalmi* Ferrer, 1998 (absent; see Ferrer 1998). Therefore, aside from species of which males have not been examined [*Platycotylusparvicollis* (Pic, 1923) and *Platycotylustenuicollis* (Fairmaire, 1893)], this new species can be distinguished from all other males of congeneric species by its long and asymmetrical epistomal horn.

The new species is most similar to *P.parvicollis*, of which a male has not yet been examined. It can be distinguished by the simple sparse punctation on the pronotum (laterally rugulose in *P.parvicollis*; see Merkl 1992) and by elytra that are scarcely striate, with elytral intervals that are neither convex nor carinate (striate in the original description of *P.parvicollis*; Pic 1923). Additionally, the smaller eyes and acutely produced temples of the new species differ from those of *P.parvicollis*.

In addition, the umbilical tubercle on the center of mentum may be one remarkable characteristic of this new species. At least, there is no such tubercle on the mentum of *P.nitidulus*, which has a small fovea in the middle.

##### Description.

Body length: 3.40 mm. Male. Elongate and flattened, shiny; dark reddish brown, head and pronotum blackish brown, elytra darkened on sutural and lateral parts.

Head obtrapezoidal, weakly convex, without frontogenal and frontoclypeal sutures; punctures coarse and dense, partly piligerous; epistome with a large asymmetrical horn in middle, distinctly emarginate on both sides of the horn, which is distinctly curved to the left and acute at its apex, with a long yellow seta arising from each emarginated anterior margin, covered with punctures; genae convex, roundly produced laterad; frons broadened, weakly convex, slightly sloping forwards, 3.83 times as wide as width of eye in lateral view; eyes entirely lateral, strongly convex laterad, without inner ocular sulci; temples slender, acutely produced laterad, setiferous and finely punctate. Antennae slender, surpassing base of elytra, almost filiform though 7^th^ antennomere dilated apicad and 8^th^ to 10^th^ ones dilated and nearly as long as wide; 11^th^ antennomere elongate. Ultimate maxillary palpomeres fusiform. Mentum transversely quadrate, weakly convex, irregularly depressed at sides, with an umbilical tubercle at middle. Submentum flat, subquadrate, strongly emarginate at sides. Gula narrow, linguiform, unevenly flat and smooth.

Pronotum obtrapezoidal, widest at apical fifth and 1.30 times as wide as long; disc slightly convex, densely punctate, punctures piligerous laterally, nearly as large as and slightly sparser than on head; anterior margin subtruncate, unbeaded; anterior corners with an acutely pointed process; lateral margins slightly rounded and evenly convergent posteriad, almost invisible in dorsal view, roundly and weakly edged at apical fifth and basal fifth, slightly sinuate before base, very finely beaded; posterior corners with processes smaller than those on anterior corners and acutely pointed laterad; basal margin weakly rounded, moderately beaded. Scutellar shield transverse, 1.67 times as wide as long, surface flat and smooth.

Elytra elongate, subparallel-sided, widest at basal sixth and 2.14 times as long as their combined width, subvertical between 7^th^ intervals and lateral margins; surface scarcely striate, with rows of punctures larger than on pronotum; intervals almost flat and impunctate; epipleura irregularly rugulose.

Prothoracic hypomera weakly depressed, with large and coarse piligerous punctures. Prosternum weakly convex, distinctly sulcate along apical bead, sparsely punctate in middle and moderately so laterally; prosternal process trapezoidal, depressed, coarsely punctate. Mesoventrite weakly convex, with large and sparse piligerous punctures. Metaventrite weakly convex, sparsely and evenly punctate though becoming denser and piligerous in each lateral fourth. Abdomen (Fig. 2A) with punctures piligerous, fine and dense; lateral margins of 3^rd^ and 4^th^ ventrites weakly and roundly produced in each apical half; 5^th^ ventrite evenly rounded at posterior margin.

Abdominal sternites VIII and IX (Fig. 2B, C); sternite VIII thin, with short setae along posterior margin; sternite IX with a pair of horizontally elongate sclerites, with an elongate protrusion on the apical third of each sclerite that is slightly curved inwards. Aedeagus (Fig. 3) lanceolate, very short, 0.11 times as long as elytra, slightly twisted towards left side of body, obsoletely margined between basale and apicale; basale 1.07 times as long as apicale; apicale rounded at apex.

Legs robust. Femora strongly dilated towards middle or distally, sparse with setiferous punctures. Tibiae short and slender; protibiae with two tibial spurs, one of which is very large and robust, curved posteriorly.

**Female**. Unknown.

##### Etymology.

The new species is dedicated to the late Dr Ottó Merkl, who made a significant contribution to the taxonomy of Tenebrionidae.

##### Distribution.

Japan: Tokara Islands (Nakanoshima Island).

##### Biological notes.

The holotype was collected by beating the dead branches of an unidentified living tree.

###### ﻿Key to species of the genus *Platycotylus* (after Merkl 1992 and Schawaller 2014)

**Table:** 

1	Pronotal surface between punctures and elytral intervals shagreened (micro-reticulated)	***P.ferrugineus* (Kaszab, 1939)**
–	Pronotal surface and elytral intervals smooth and shiny	**2**
2	Antennomere 11 elongate, at least 3 times longer than wide, pronotum flat	***P.tenuicornis* (Fairmaire, 1893)**
–	Antennomere 11 only 2 times longer than wide, pronotum convex	**3**
3	Pronotum more transverse, with distinctly prominent anterolateral corners	***P.nitidulus* (MacLeay, 1872)**
–	Pronotum less transverse, subquadrate or trapezoidal, with short anterolateral corners	**4**
4	Pronotum longer, trapezoidal, elytral interval 7 convex	*P.palmi* (Ferrer, 1998)
–	Pronotum subquadrate or obtrapezoidal, elytral interval 7 keeled	**5**
5	Eyes moderate in size, temple not produced, pronotum laterally with rugulose punctation, elytra striate	***P.parvicollis* (Pic, 1923)**
–	Eyes smaller, temple acutely produced, pronotum laterally with simple sparse punctation, elytra scarcely striate	***P.merkli* sp. nov.**

###### ﻿Abdominal pits and male genital morphology

Matthews and Bouchard (2008) regarded the abdominal pits of the palorine male and the inverted aedeagus as two autapomorphies of Palorini. However, the abdominal pits were absent on the males of *Platycotylus* examined by Masumoto and Grimm (2004). The male of *P.merkli* sp. nov. also does not possess these pits, indicating that they are lacking in the genus *Platycotylus*.

The male genital structures of *Platycotylus* have been poorly studied only in two species: *P.palmi* by Ferrer (1988) and *P.nitidulus*, with a simple illustration by Matthews and Bouchard (2005). The shapes of the aedeagi of these species are similar to each other and inverted, although the orientation of the aedeagus of *P.palmi* has yet to be examined. Nevertheless, the shape of the aedeagus of *P.merkli* sp. nov. is lanceolate, twisted in the middle, and with the basale slightly longer than the apicale (Fig. 3). Thus, the genital morphology of *P.merkli* sp. nov. is substantially different from that of its congeners, which highlights the systematic peculiarity of this new species.

A similar pattern of variation in male genital morphology is shown in a lineage of Erotylidae (Cucujoidea), which is mainly found on the male cones of cycad plants and contains three genera: *Cycadophila* Xu, Tang & Skelley, 2015, *Pharaxonotha* Reitter, 1875, and *Ceratophila* Tang, Skelley & Pérez-Farrera, 2018. Males of *Cycadophila* possess an aedeagus that is twisted towards the left side, whereas the other genera possess inverted male genitalia (Xu et al. 2015; Tang et al. 2020). Tang et al. (2020) suggested that these shapes and orientations of the genitalia may be related to the mating position (side-to-side or end-to-end) as an adaptation to mating in the tight spaces of cycad cones. These authors also indicated that these morphological adaptations had evolved independently in each genus. The twisted aedeagus of the new species described here, as well as the inverted aedeagus of the tribe in general, may be associated with the habitat of the insect, i.e., under the hardly loosened bark of dead branches. Further studies on these habitats, as well as on the mating behavior and genital morphology of Palorini, are required.

## Supplementary Material

XML Treatment for Platycotylus merkli
